# Relationship of VDR and VDBP gene polymorphisms with sepsis susceptibility and prognosis

**DOI:** 10.3389/fgene.2025.1590750

**Published:** 2025-04-25

**Authors:** Chan Lian, Kangtai Ying, Haiyan Shao, Haiting Gu, Wenwei Mao

**Affiliations:** Department of Respiratory Medicine, The First People’s Hospital of Wenling City, Wenling, Zhejiang, China

**Keywords:** sepsis, vitamin D receptor, vitamin D binding protein, gene polymorphism, susceptibility, prognosis

## Abstract

**Objective:**

This research focused on the association of vitamin D receptor (VDR) and vitamin D binding protein (VDBP) gene polymorphisms with sepsis susceptibility and prognosis.

**Methods:**

110 septic patients were selected as the sepsis group, and another 100 patients with common infections who did not develop sepsis as the control group. 28 days death of patients in the sepsis group were counted. Within 24 h of admission, patients were scored by Acute Physiology and Chronic Health Evaluation Ⅱ (APACHE Ⅱ) and Sequential Organ Failure Assessment (SOFA). Serum lactate (Lac), C-reactive protein (CRP), procalcitoninogen (PCT) and vitamin D levels were evaluated. All patient DNAs were extracted. The polymorphisms of VDR and VDBP genes and vitamin D genes *Fok Ⅰ (rs2228570)* and VDBP *rs4588* locus were tested and compared in both groups; and the receiver operating characteristic (ROC) curves were plotted to calculate the area under the area under the curve (AUC) and assess the diagnostic value of each indicator for sepsis. Patients in the sepsis group were categorized into a death group and a survival group, the above indicators were compared in the two groups and the factors affecting the prognosis of sepsis patients were analyzed.

**Results:**

Compared to the control group, the sepsis group exhibited higher APACHE II scores, SOFA scores, serum Lac, CRP, PCT levels, VDR *Fok Ⅰ (rs2228570)* locus *f allele* and VDBP *rs4588* locus *A allele* frequencies, and lower vitamin D levels (*P* < 0.05). The ROC curve analysis showed that the AUC for the diagnosis of sepsis using the *AA genotype* at the VDBP gene *rs4588* locus was 0.579 (95% CI: 0.501–0.656) (sensitivity: 52.70%; specificity: 63.00%, *P* < 0.05). APACHE II and SOFA scores and serum levels of Lac, CRP, and PCT in the death group were raised and vitamin D levels were diminished than those in the survival group (*P* < 0.05). Raised APACHE II and SOFA scores were independent risk factors affecting sepsis prognosis.

**Conclusion:**

The f allele at the VDR *Fok Ⅰ (rs2228570)* locus and the *A allele* at the VDBP *rs4588* locus significantly raise the risk of sepsis in patients.

## Introduction

Sepsis is a clinical syndrome caused by infection-induced dysregulation of the host response, resulting in severe organ function damage, which is a serious disease with a very high mortality rate and is the leading cause of death due to infection (Sheng et al., 2021). This extremely complex and difficult-to-treat disorder can occur at any age and under any underlying disorder (Qi et al., 2023). Genetic variations have been shown to link differences in the death of sepsis patients. Single nucleotide polymorphisms are one of the most common genetic variants in humans and are closely associated with genetic susceptibility, early diagnosis, disease progression and prognosis of sepsis (Sheng et al., 2021).

The vitamin D receptor (VDR), a member of the nuclear receptor superfamily, performs a central role in the biological actions of vitamin D. The VDR regulates calcium/phosphate homeostasis, cell proliferation and differentiation, and the expression of numerous genes in the immune response, primarily in a ligand-dependent manner (Wang et al., 2012). In higher vertebrates, VDR is the only known regulatory mediator of the hormone 1,25-dihydroxyvitamin D3 [1,25(OH)2D3]. It acts in the nucleus of vitamin D target cells to regulate the expression of genes whose products control a wide range of cell type-specific biological functions (Pike et al., 2017). Data suggest that two variants of the VDR gene, *rs2107301* and *rs2189480*, perform a predominant part in susceptibility to sepsis in children (He et al., 2021); mutations in the *rs2107301-C* and *rs2189480-C alleles* are concerned with a reduced risk of sepsis (He et al., 2021); and *G>T* at the *rs739837* locus of the VDR gene is concerned with a neonatal sepsis risk reduction (Xiao L. et al., 2022). The VDR gene has many different polymorphisms. Some of these polymorphisms might affect VDR function, such as the *FokI (rs2228570, T/C)* single nucleotide polymorphism (Li et al., 2023). The *Fok I (rs2228570)* single nucleotide polymorphism (SNP) of the VDR gene is located in exon 2 and is characterized by a nucleotide substitution of thymine (T) to cytosine (C). This substitution alters the position of the original translation initiation codon, causing initiation of translation to occur earlier, shifting from the first ATG codon in the wild-type protein to an upstream ACG (which becomes a functional initiation site due to the influence of the *C allele*), resulting in the encoded VDR protein being shortened from 427 amino acids to 424 amino acids. This shorter VDR protein isoform exhibits enhanced transcriptional activation ability, which may influence the biological functions of VDR and its role in relevant physiological and pathological processes (Agliardi et al., 2023). The VDR *Fok I (rs2228570)* polymorphism has been strongly associated with susceptibility to sepsis, and patients with sepsis have lower levels of 25-hydroxyvitamin D (25(OH)D). Mutations in the VDR *Fok I (rs2228570)* gene may alter sepsis risk (Li et al., 2023).

On the other hand, Vitamin D binding protein (VDBP) is a major carrier of vitamin D (Fernando et al., 2020) and a key node in the regulation of the vitamin D system (Gauzzi, 2018). As the main plasma carrier of vitamin D metabolites, VDBP regulates the stability and bioavailability of vitamin D metabolites (Gauzzi, 2018). VDBP can mediate a variety of human biological processes (Cho et al., 2019). A previous study has reported that serum VDBP levels are linked to 30-d mortality, and serum VDBP levels are higher in survivors versus non-survivors in terms of 30-d mortality in patients with sepsis (Yoo et al., 2020). VDBP is expressed at low levels in sepsis. There are data that polymorphisms or variants in the gene locus *rs7401* (mutant) result in decreased levels of VDBP and vitamin D, and that decreased levels of vitamin D are associated with mortality in patients with sepsis (Kahar et al., 2023); *rs4588* is one of the most common polymorphisms in the VDBP gene. *rs4588* might possess a correlation with differences in serum vitamin D status and vitamin D metabolites (Rozmus et al., 2022).

Given the potential role of VDR and VDBP gene polymorphisms in the pathogenesis of sepsis, an in-depth exploration of the relationship between these gene polymorphisms and sepsis susceptibility and prognosis not only helps to elucidate the genetic susceptibility mechanisms of sepsis but may also provide new molecular targets for the early diagnosis, risk assessment, and personalized treatment of sepsis. Therefore, this study aims to systematically analyze the correlation between VDR and VDBP gene polymorphisms and sepsis susceptibility and prognosis, with the goal of providing a scientific basis for precision medicine in sepsis.

## Materials and methods

### Ethics statement

The study was ratified by the Medical Ethics Committee of The First People’s Hospital of Wenling City and the written informed consent was acquired from all participants.

### Study subjects and grouping

A total of 110 septic patients admitted to The First People’s Hospital of Wenling City from November 2019 to November 2022 were included in the sepsis group, and they were separated into the survival group and the death group based on different prognosis. Inclusion criteria: Patients diagnosed with sepsis according to the Sepsis 3.0 international standards (Singer et al., 2016); patients meeting national and international clinical diagnosis criteria for sepsis; those whose age >18 years; those informed about the study. Exclusion criteria: patients with a history of special medication (e.g., anticoagulants, immunosuppressants, hormonal drugs, etc.); those suffering from autoimmune diseases; pregnant and lactating women; those combined with malignant tumors; those underwent radiotherapy; those suffered acute poisoning. In addition, 100 patients with common infections (without sepsis) admitted to The First People’s Hospital of Wenling City within the same period were included as the control group, and those with infections except sepsis were included, with the same exclusion criteria as those in the sepsis group.

### Data collection

Baseline information of patients at the time of admission, including age, gender, smoking, alcohol consumption, diabetes, and hypertension history, was collected through the electronic medical record. The 28 days death of the patients in the sepsis group during hospitalization were counted. Within 12 h of admission, patients were assessed using the Acute Physiology and Chronic Health Evaluation II (APACHE II) scoring system and the Sequential Organ Failure Assessment (SOFA) score. Laboratory indicators, including blood lactate (Lac), C-reactive protein (CRP), and procalcitonin (PCT), were also measured.

### Laboratory indicator detection

Within 24 h of admission, 3 mL of fasting elbow venous blood was drawn from the patients and centrifuged at 3,000r/min for 10 min to obtain serum. Serological indicators (CRP, PCT, and Lac) were detected. PCT was measured using chemiluminescence (kit provided by Shenzhen Mindray Bio-Medical Electronics Co., Ltd.), Lac was measured using colorimetry (kit provided by Beijing Biolab Technology Co., Ltd.), and CRP was measured using immunoturbidimetry (kit purchased from Shanghai Jingkang Bioengineering Co., Ltd.).

### Serum 25(OH)D assay

After the patients were admitted, 3–5 mL of the venous blood was collected within 24 h. Serum 25(OH)D levels (ng/mL) were measured utilizing the cobas e 602 electrochemiluminescence analyzer (Roche, Germany).

### Gene polymorphism assay

Polymerase Chain Reaction-Restriction Fragment Length Polymorphism (PCR-RFLP) was used for genotype VDR and VDBP genes. After enrollment, 5 mL of fasting venous blood was collected from all patients using anticoagulant tubes, and genomic DNA was extracted using a whole blood DNA extraction kit (QIAGEN, Germany). The *rs2228570* and *rs4588* loci were identified as SNPs for VDR and VDBP genotype analysis using the SNP picker program. Primers were designed using Primer Premier 5.0 and synthesized by Beijing Bio-Tech Corporation. The primer sequences are as follows: for the *Fok I (rs2228570)* locus, the forward primer is 5′-TGA​CTC​TGG​CTC​TGA​CCG-3′, and the reverse primer is 5′-AAG​ACC​CTC​CTG​CTC​CTG-3'. For the *rs4588* locus, the forward primer is 5′-GAA​GAG​AAG​GTG​AAA​GGT​TAG​G-3′, and the reverse primer is 5′-CCT​GTC​ACA​TAA​TGG​CAT​CTC​AAT-3'. PCR amplification was performed according to the PCR procedure, with the reaction conditions being 5 min of pre-denaturation at 94°C, followed by 35 cycles of 30 s of denaturation at 94°C, 30 s of annealing at 60°C, and 30 s of extension at 72°C, and then 7 min of extension at 72°C. The PCR products were digested with *Fok Ⅰ* and *Sty Ⅰ* restriction endonucleases [both purchased from Fermentas (FassDigest)], and the digestion products were subjected to agarose gel electrophoresis. The bands were observed, photographed, recorded, and analyzed using a GD-1000 gel imaging analyzer for genotyping.

### Statistics

SPSS20.0 statistical software was applied for data processing. Numeration data were expressed as a rate (%), and the comparison between groups was performed by the chi-square test or Fisher’s exact test. Measurement data were depicted as mean ± standard deviation ( $\bar{x}$ ± s), and comparisons between groups were performed using the independent sample t-test. Multivariate logistic regression was used to analyze the factors influencing the prognosis of sepsis patients. Receiver operating characteristic (ROC) curves were plotted, and the area under the curve (AUC) was calculated to assess the diagnostic value of each indicator for sepsis. Data were statistically analyzed using two-tailed tests. The difference of *P* < 0.05 was considered as having a statistical significance.

## Results

### General data

No difference was found between the two groups in terms of age, gender, and history of smoking, drinking, diabetes, and hypertension between the control group and sepsis group (*P* > 0.05). The sepsis group possessed elevated APACHE Ⅱ and SOFA scores, and serum Lac, CRP, and PCT levels, and lower 25(OH)D levels versus the control group (*P* < 0.05) (Table 1).

**TABLE 1:** General data.

Indicators	The control group (n = 100)	The sepsis group (n = 110)	P Value
Age (years)	59.20 (SD 10.17)	58.32 (SD 10.26)	0.534
Gender (cases)			0.119
Male	55	73	
Female	45	37	
Smoking history (cases)			0.890
Yes	57	61	
No	43	49	
Drinking history (cases)			>0.999
Yes	50	54	
No	50	56	
Diabetes (cases)			0.778
Yes	37	43	
No	63	67	
Hypertension (cases)			0.677
Yes	45	46	
No	55	64	
Site of infection (cases)			
Lung	50	58	
Abdomen	32	37	
Others	18	15	
28-d mortality (cases)	0	31	<0.001
APACHE Ⅱ scores (points)	13.16 (SD 3.37)	15.47 (SD 4.36)	<0.001
SOFA scores (points)	4.79 (SD 1.64)	6.94 (SD 2.44)	<0.001
Lac (mmol/L)	1.42 (SD 0.12)	2.46 (SD 0.29)	<0.001
CRP (mg/L)	76.15 (SD 7.45)	145.30 (SD 14.42)	<0.001
PCT (g/L)	9.61 (SD 2.66)	47.40 (SD 10.36)	<0.001
25(OH) D (ng/mL)	21.64 (SD 3.88)	6.74 (SD 2.06)	<0.001

### Distribution of genotypes and allele frequency at the VDR *Fok Ⅰ (rs2228570)* locus

The genotype distribution of the VDR *Fok Ⅰ (rs2228570)* locus in septic patients conformed to the Hardy-Weinberg equilibrium (*P* > 0.05), which meant that the study population of this experiment was representative of the population.

In the sepsis group, 7 cases of *FF* genotype, 42 cases of *Ff* genotype, and 61 cases of *ff* genotype were detected at the VDR *Fok I (rs2228570)* locus. In the control group, 16 cases of *FF* genotype, 39 cases of *Ff* genotype, and 45 cases of *ff* genotype were detected at the same locus. The frequency of the *f* allele at the VDR gene *Fok I (rs2228570)* locus was higher in the sepsis group than in the control group (*P* < 0.05). The frequency of the *Ff* + *ff* genotype at the VDR *Fok I (rs2228570)* locus was higher in the sepsis group than in the control group (*P* < 0.05), while the frequency of the *FF* genotype was lower in the sepsis group than in the control group (*P* < 0.05) (Table 2).

**TABLE 2:** Distribution of genotypes and allele frequency at the VDR *Fok Ⅰ (rs2228570)* locus [case (%)].

Grouping	Genotype			Allele	
	*FF*	*Ff*	*ff*	*F*	*f*
The control group (n = 100)	16 (16.00%)	39 (39.00%)	45 (45.00%)	71 (35.50%)	129 (64.50%)
The sepsis group (n = 110)	7 (6.36%)	42 (38.18%)	61 (55.45%)	56 (25.45%)	164 (74.55%)
*P* value	0.029	>0.999	0.167	0.026	

### Distribution of genotypes and allele frequency at the VDBP *rs4588* locus

In this experiment, the genotype distribution of the VDBP *rs4588* locus in septic patients conformed to the Hardy-Weinberg equilibrium (*P* > 0.05), which revealed that the study subjects of this experiment could represent the population.

In the sepsis group, 9 cases of *CC* genotype, 43 cases of *CA* genotype, and 58 cases of *AA* genotype were detected at the VDBP *rs4588* locus. In the control group, 12 cases of *CC* genotype, 51 cases of *CA* genotype, and 37 cases of *AA* genotype were detected at the same locus. The frequency of the *A* allele at the VDBP gene *rs4588* locus was higher in the sepsis group than in the control group (*P* < 0.05). The frequency of the *AA* genotype at the VDBP *rs4588* locus was higher in the sepsis group than in the control group (*P* < 0.05) (Table 3).

**TABLE 3:** Distribution of genotypes and allele frequency at the VDBP *rs4588* locus [case (%)].

Grouping	Genotype			Allele	
	*CC*	*CA*	*AA*	*C*	*A*
The control group (n = 100)	12 (12.00%)	51 (51.00)	37 (37.00%)	75 (37.50%)	125 (62.50%)
The sepsis group (n = 110)	9 (8.18%)	43 (39.09%)	58 (52.73%)	61 (27.73%)	159 (72.27%)
*P* value	0.369	0.096	0.027	0.037	

### ROC curve analysis of the predictive value of VDR and VDBP gene polymorphisms for sepsis

**FIGURE 1:**
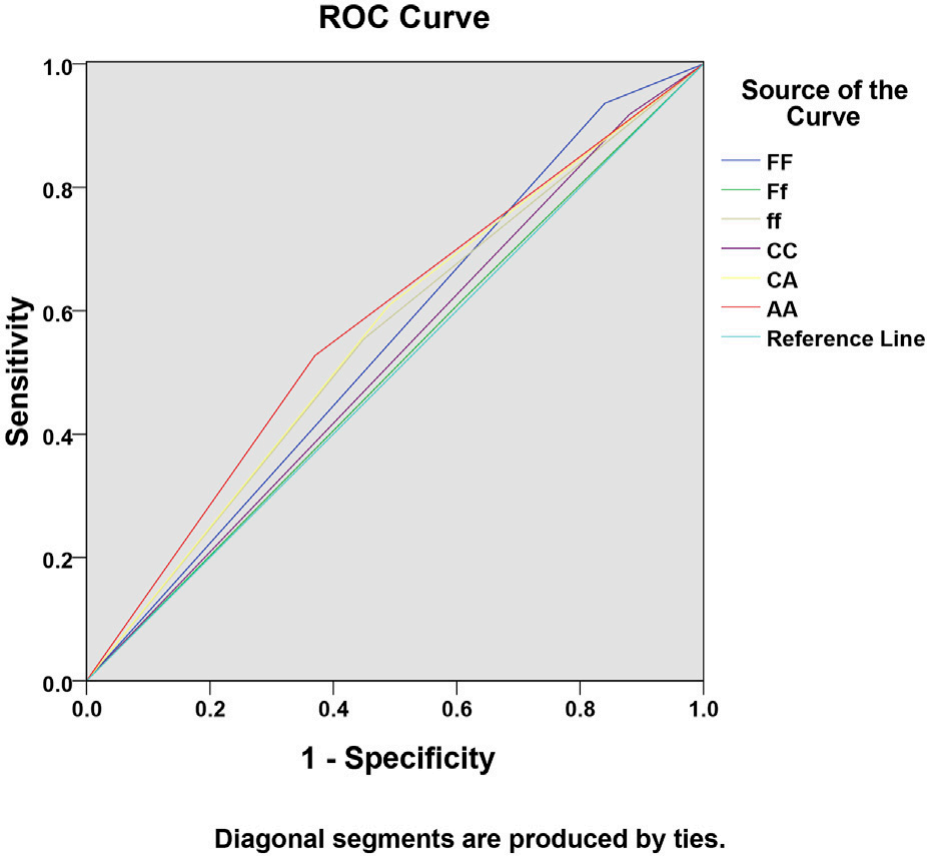
ROC curve showing the predictive value of VDR and VDBP gene polymorphisms for sepsis.

The ROC curve analysis results showed that the AUC for diagnosing sepsis with the *AA* genotype at the VDBP gene *rs4588* locus was 0.579 (95% CI: 0.501∼0.656), with a sensitivity of 52.70% and a specificity of 63.00% (*P* < 0.05) (Table 4; Figure 1).

**TABLE 4:** Predictive efficacy of VDR and VDBP gene polymorphisms for sepsis.

Item	AUC	95%CI	*P* Value	Sensitivity/%	Specificity/%	Youden index
VDR gene *Fok I (rs2228570)* locus						
*FF* genotype	0.548	0.470∼0.626	0.228	93.60	16.00	0.096
*Ff* genotype	0.504	0.426∼0.582	0.919	61.80	39.00	0.008
*ff* genotype	0.552	0.474∼0.630	0.191	55.50	55.00	0.105
VDBP gene *rs4588* locus						
*CC* genotype	0.519	0.441∼0.598	0.633	91.80	12.00	0.038
*CA* genotype	0.56	0.482∼0.637	0.136	60.90	51.00	0.119
*AA* genotype	0.579	0.501∼0.656	0.049	52.70	63.00	0.157

### Clinical characteristics of septic patients in the survival and death groups

No difference was found between the two groups in terms of age, gender, smoking, drinking, diabetes, and hypertension history (*P* > 0.05). APACHE Ⅱ and SOFA scores, and serum Lac, CRP, and PCT levels in the death group were elevated versus those in the survival group, and the 25(OH)D levels were lower versus that in the survival group (*P* < 0.05) (Table 5).

**TABLE 5:** Clinical characteristics of septic patients in the surviving and dying groups.

Indicators	The survival group (n = 79)	The death group (n = 31)	P Value
Age (years)	57.88 (SD 10.73)	59.44 (SD 9.01)	0.475
Gender (cases)			0.269
Male	55	18	
Female	24	13	
Smoking history (cases)			0.288
Yes	41	20	
No	38	11	
Drinking history (cases)			0.091
Yes	43	11	
No	36	20	
Diabetes (cases)			0.278
Yes	28	15	
No	51	16	
Hypertension (cases)			>0.999
Yes	33	13	
No	46	18	
APACHE Ⅱ score (points)	13.39 (SD 3.05)	20.78 (SD 2.09)	<0.001
SOFA score (points)	5.77 (SD 1.71)	9.91 (SD 1.17)	<0.001
Lac (mmol/L)	2.32 (SD 0.20)	2.81 (SD 0.14)	<0.001
CRP (mg/L)	138.41 (SD 10.09)	162.85 (SD 6.92)	<0.001
PCT (g/L)	42.45 (SD 7.25)	60.01 (SD 4.97)	<0.001
25(OH) D (ng/mL)	7.70 (SD 1.52)	4.30 (SD 0.94)	<0.001

### Multivariate analysis of the prognosis of septic patients

Increased APACHE II and SOFA scores were independent risk factors that affected the prognosis of patients with sepsis (*P* < 0.05) (Table 6).

**TABLE 6:** Multivariate analysis of the prognosis of septic patients.

Factors	β	SE	Wals	P	OR	95%CI
APACHE Ⅱ score	0.994	0.36	7.63	0.006	2.702	1.335∼5.47
SOFA score	1.174	0.465	6.38	0.012	3.235	1.301∼8.043

## Discussion

Sepsis refers to clinical syndromes resulting from the host reaction infection-induced disorder, which can result in severe organ function damage (Sheng et al., 2021). Sepsis is a severe disorder with a high rate of death. Therefore, it is of great significance to address the correlation between gene polymorphisms and sepsis susceptibility and prognosis. This study focused on the correlation of VDR and VDBP gene polymorphisms with sepsis susceptibility and prognosis.

Through literature review, we found that VDR-*Fok I* polymorphism is associated with susceptibility to rheumatoid arthritis in the European population (Song et al., 2016). In the Arab population, VDR *Fok I* polymorphism is related to susceptibility to systemic lupus erythematosus (Bae and Lee, 2018). Additionally, the VDBP (*rs4588*) *T/T* genotype is a susceptibility factor for anterior septal myocardial infarction (Persic et al., 2019). The associations between these different diseases and VDR and VDBP gene polymorphisms suggest that these genes may play important roles in the occurrence and development of various diseases. As previously reported, the VDR gene might function as a sepsis-susceptibility gene (He et al., 2021). A previous study has demonstrated that VDR is linked to intestinal barrier damage in sepsis. VDR expression levels are lower in sepsis, and VDR upregulation has the ability to diminish intestinal barrier damage in sepsis, which may act as a potential biomarker and treatment target in sepsis (Shang et al., 2023). Growing evidence has reported that VDR polymorphism has the capacity to modulate the expression levels of VDR that significantly influence susceptibility and immunity to microbial infections (Tayel et al., 2018). It is also explored the correlation of VDR gene polymorphisms with sepsis risk and death in a previous study. VDR *Fok I* possibly plays a role in raising sepsis risk, as a molecular biomarker (Zeljic et al., 2017). Both *Fok I* variants and minor alleles are more prevalent in patients with sepsis. Moreover, VDR *Fok I* variants are demonstrated to be predisposed to susceptibility to sepsis (Yang et al., 2022). *Fok I Ff* and *ff* genotypes are linked to elevated risk and frequency of bronchopulmonary dysplasia (BPD). In addition, variant *Fok I* genotype can raise BPD risk independent of sepsis (Koroglu et al., 2014). Recent evidence also suggests that variants in the VDR genes, particularly the *rs2228570* (*Fok I*) “*f*” allele and the *rs7975232* (*Apa I*) “aa” genotype, are markers of poor prognosis (Kotur et al., 2023).

The results of this paper unveiled that the sepsis group had a higher frequency of the f allele at the *Fok I* (*rs2228570*) locus of the VDR gene in contrast with the control group. This indicates that the f allele may be associated with increased susceptibility to sepsis. The *f* alleles may lead to alterations in the structure or function of the VDR, which in turn affects the signaling and bioactivity of vitamin D *in vivo*. Meanwhile, vitamin D acts an important role in immunomodulation, and changes in its level may affect the body’s immune response to infection (Gauzzi, 2018), thereby increasing the risk of sepsis. In addition to this, the study also revealed a higher frequency of the *Ff* + *ff* genotype at the *Fok I* (*rs2228570*) locus of the VDR in the sepsis group compared to the control group. This further supports the association between the *f* allele and susceptibility to sepsis. The *Ff* + *ff* genotype may carry more *f* alleles, leading to further alter rations in VDR function, which may affect the body’s immune response to infection. Xinyue Yang et al. revealed that *Fok I* variants and minor alleles are more prevalent in sepsis patients compared to healthy controls. Furthermore, the combined plasma levels of 25(OH) vitamin D and *FokI* polymorphism play an important role in susceptibility to sepsis and septic shock. They indicated that the *Fok I* mutation is associated with an increased risk of sepsis and septic shock (Yang et al., 2022). J Rathored et al. reported in their study that the *FokI Ff* genotype is positively correlated with multidrug-resistant tuberculosis; in both tuberculosis groups, the *Ff* genotype and *f* allele frequencies of *Fok I* are higher (Rathored et al., 2012).

VDBP performs multiple functions as a transporter for various ligands and is involved in many systemic and local physiological and pathological processes (Lisowska-Myjak et al., 2020). Data demonstrate that VDBP expression is low in sepsis. VDBP may be protective against sepsis-induced liver injury (Xiao K. et al., 2022). *rs4588* may be related to the differences in serum vitamin D status (Rozmus et al., 2020). Data disclose that the VDBP (*rs4588*) *T/T* genotype is a susceptibility factor for anterior septal myocardial infarction (Persic et al., 2019); the *A* allele of the *rs4588* is connected with lower VDBP and bone mineral density compared to the *CC* allele (Rivera-Paredez et al., 2021). Our study unearthed that the frequency of the *A* allele at the *rs4588* locus of the VDBP gene was higher in the sepsis group in comparison with in the control group. This implies that the A allele may be connected with increased susceptibility to sepsis. VDBP is a major binding protein for vitamin D and is involved in the transport and distribution of vitamin D in the body (Gauzzi, 2018) (Cho et al., 2019). The *A* allele may lead to changes in the structure or function of VDBP, which may affect the utilization of vitamin D and the immunomodulatory effects, thereby increasing the risk of sepsis. Also, the findings observed a higher frequency of the *AA* genotype at the *rs4588* locus of the VDBP in the sepsis group versus that in the control group, which further strengthens the association between the *A* allele and susceptibility to sepsis. The *AA* genotype may carry 2 *A* alleles, resulting in significant alterations in the function of the VDBP, which further affects vitamin D metabolism and immune regulatory functions. It has been reported that subjects with *rs4588 AA* or *AC* genotypes exhibit significantly higher levels of low-density lipoprotein compared to those with the *CC* genotype. The *rs4588 AC* genotype and *A* allele are associated with an increased risk of Polycystic Ovary Syndrome and infertility (Akbari et al., 2024). Notably, Jolanta et al. confirmed that the *rs4588-A* allele and its corresponding haplotype have a protective effect on multiple sclerosis (Kalnina et al., 2025). We further analyzed the predictive value of VDR and VDBP gene polymorphisms for sepsis. The results indicated that the VDBP gene *rs4588 AA* genotype has a certain predictive value for sepsis.

VDR is one of the significant predictors of 28-d death, which can predict poor results in sepsis patients, and low VDR levels are involved in reduced overall survival in sepsis patients (Erdogan and Findikli, 2021). Furthermore, VDR levels have a negative correlation with Lac, CRP, APACHE II scores and SOFA scores in sepsis patients (Erdogan and Findikli, 2021). Lower serum levels of VDBP are involved in raised APACHE II and SOFA scores, indicating that VDBP may be protective against sepsis-induced diseases (Xiao K. et al., 2022). Bio-available 25(OH)D can act as a mediator or biomarker of negative results among acute kidney injury patients (Leaf et al., 2013). 25(OH)D plasma levels and *Fok I* polymorphisms in combination can reveal a crucial role in sepsis and septic shock predisposition. It is also reported that low 25(OH)D levels and *Fok I* mutants have an association with an elevated risk of sepsis and septic shock. Lower 25(OH)D levels are high-prevalent in patients with sepsis (Yang et al., 2022). In our paper, it was found that the sepsis group possessed higher APACHE Ⅱ scores, SOFA scores, and serum Lac, CRP, and PCT levels, and lower 25(OH)D levels versus the control group; the death group had higher APACHE Ⅱ scores, SOFA scores, and serum Lac, CRP, and PCT levels, and lower 25(OH)D levels versus the survival group. Furthermore, we also found that elevated APACHE II and SOFA scores were independent risk factors affecting the prognosis of sepsis patients. High APACHE II and SOFA scores indicate that patients are at risk for severe organ dysfunction and poor prognosis. Gene polymorphisms may further exacerbate organ damage and dysfunction by influencing the immune response and inflammatory response. Low 25(OH)D levels may be associated with the absence of an important role for vitamin D in immune regulation, further affecting patient prognosis. Gene polymorphisms may increase the risk of patient mortality by affecting the metabolism and utilization of vitamin D and reducing its immunomodulatory effects.

In summary, this research demonstrates that the f allele at the VDR *Fok I* (*rs2228570*) locus and the *A* allele at the VDBP *rs4588* locus significantly elevated the risk of developing sepsis in patients after severe trauma, and raised APACHE II and SOFA scores were independent risk factors affecting the prognosis of sepsis patients. This study provides a reference value for studying the relationship of VDR and VDBP gene polymorphisms with sepsis susceptibility and prognosis. However, this study did not perform sample size calculation, and the sample size was limited, which may affect the generalizability of the findings. Additionally, this study did not further explore the correlation between VDR and VDBP gene polymorphisms and the severity of sepsis, which is also a limitation of our study. Future research should prioritize sample size calculation to provide a more solid foundation for the study and further explore the specific mechanisms of these gene polymorphisms, opening up new avenues for the prevention and treatment of sepsis.

## Data Availability

The original contributions presented in the study are included in the article/supplementary material, further inquiries can be directed to the corresponding author.

## References

[B1] Agliardi C. Guerini F. R. Bolognesi E. Zanzottera M. Clerici M. (2023). VDR gene single nucleotide polymorphisms and autoimmunity: a narrative review. Biol. (Basel) 12 (7), 916. 10.3390/biology12070916 PMC10376382 37508347

[B2] Akbari L. N. Kheirollahi A. Vatannejad A. Hamidi H. (2024). Association of rs4588 polymorphism in vitamin D binding protein gene with polycystic ovarian syndrome in Iranian women: a case-control study. BMC Res. Notes 17 (1), 207. 10.1186/s13104-024-06857-x 39068475 PMC11283716

[B3] Bae S. C. Lee Y. H. (2018). Vitamin D receptor FokI, TaqI, and ApaI polymorphisms and susceptibility to systemic lupus erythematosus: an updated meta-analysis. Clin. Rheumatol. 37 (6), 1529–1537. 10.1007/s10067-018-4036-z 29468338

[B4] Cho M. C. Kim J. H. Jung M. H. Cho I. A. Jo H. C. Shin J. K. (2019). Analysis of vitamin D-binding protein (VDBP) gene polymorphisms in Korean women with and without endometriosis. Clin. Exp. Reprod. Med. 46 (3), 132–139. 10.5653/cerm.2019.00122 31405270 PMC6736509

[B5] Erdogan M. Findikli H. A. (2021). Novel biomarker for predicting sepsis mortality: vitamin D receptor. J. Int. Med. Res. 49 (8), 3000605211034733. 10.1177/03000605211034733 34396836 PMC8371733

[B6] Fernando M. Ellery S. J. Marquina C. Lim S. Naderpoor N. Mousa A. (2020). Vitamin D-binding protein in pregnancy and reproductive Health. Nutrients 12 (5), 1489. 10.3390/nu12051489 32443760 PMC7285222

[B7] Gauzzi M. C. (2018). Vitamin D-binding protein and multiple sclerosis: evidence, controversies, and needs. Mult. Scler. 24 (12), 1526–1535. 10.1177/1352458518792433 30113253

[B8] He D. Lu X. Li W. Wang Y. Chen Y. Liu W. (2021). Vitamin D receptor is a sepsis-susceptibility gene in Chinese children. Med. Sci. Monit. 27, e932518. 10.12659/MSM.932518 34689148 PMC8552509

[B9] Kahar L. A. Yusrawati Y. Jamsari J. Maskoen T. Aribowo K. Sari W. M. (2023). Vitamin D-binding protein and the role of its gene polymorphisms in the mortality of sepsis patients. Acta Med. Acad. 52 (3), 212–220. 10.5644/ama2006-124.428 38407088 PMC10945317

[B10] Kalnina J. Trapina I. Plavina S. Leonova E. Paramonovs J. Sjakste N. (2025). Search for disease-specific genetic markers originated from the vitamin D binding protein gene polymorphisms in the multiple sclerosis cohort in the Latvian population. Int. J. Mol. Sci. 26 (6), 2555. 10.3390/ijms26062555 40141197 PMC11941955

[B11] Koroglu O. A. Onay H. Cakmak B. Bilgin B. Yalaz M. Tunc S. (2014). Association of vitamin D receptor gene polymorphisms and bronchopulmonary dysplasia. Pediatr. Res. 76 (2), 171–176. 10.1038/pr.2014.63 24796371

[B12] Kotur N. Stankovic B. Pavlovic S. (2023). Micronutrients, genetics and COVID-19. Curr. Opin. Clin. Nutr. Metab. Care 26 (4), 309–315. 10.1097/MCO.0000000000000942 37144461

[B13] Leaf D. E. Waikar S. S. Wolf M. Cremers S. Bhan I. Stern L. (2013). Dysregulated mineral metabolism in patients with acute kidney injury and risk of adverse outcomes. Clin. Endocrinol. (Oxf) 79 (4), 491–498. 10.1111/cen.12172 23414198 PMC3686895

[B14] Li Q. Li W. Chen M. Chai Y. Guan L. Chen Y. (2023). Association of vitamin D receptor gene polymorphism with the risk of sepsis: a systematic review and meta-analysis. Med. Baltim. 102 (38), e35130. 10.1097/MD.0000000000035130 PMC10519506 37746941

[B15] Lisowska-Myjak B. Jóźwiak-Kisielewska A. Łukaszkiewicz J. Skarżyńska E. (2020). Vitamin D-binding protein as a biomarker to confirm specific clinical diagnoses. Expert Rev. Mol. Diagn 20 (1), 49–56. 10.1080/14737159.2020.1699064 31795772

[B16] Persic V. Raljević D. Markova-Car E. Cindrić L. Miškulin R. Žuvić M. (2019). Vitamin D-binding protein (rs4588) T/T genotype is associated with anteroseptal myocardial infarction in coronary artery disease patients. Ann. Transl. Med. 7 (16), 374. 10.21037/atm.2019.07.49 31555688 PMC6736812

[B17] Pike J. W. Meyer M. B. Lee S. M. Onal M. Benkusky N. A. (2017). The vitamin D receptor: contemporary genomic approaches reveal new basic and translational insights. J. Clin. Invest 127 (4), 1146–1154. 10.1172/JCI88887 28240603 PMC5373853

[B18] Qi A. Liu Y. Zhai J. Wang Y. Li W. Wang T. (2023). RNF20 deletion causes inflammation in model of sepsis through the NLRP3 activation. Immunopharmacol. Immunotoxicol. 45 (4), 469–478. 10.1080/08923973.2023.2170241 36650938

[B19] Rathored J. Sharma S. K. Singh B. Banavaliker J. N. Sreenivas V. Srivastava A. K. (2012). Risk and outcome of multidrug-resistant tuberculosis: vitamin D receptor polymorphisms and serum 25(OH)D. Int. J. Tuberc. Lung Dis. 16 (11), 1522–1528. 10.5588/ijtld.12.0122 22990231

[B20] Rivera-Paredez B. Hidalgo-Bravo A. León-Reyes G. Antuna-Puente B. Flores Y. N. Salmerón J. (2021). Association of GC variants with bone mineral density and serum VDBP concentrations in Mexican population. Genes (Basel) 12 (8), 1176. 10.3390/genes12081176 34440350 PMC8391993

[B21] Rozmus D. Ciesielska A. Płomiński J. Grzybowski R. Fiedorowicz E. Kordulewska N. (2020). Vitamin D binding protein (VDBP) and its gene polymorphisms-the risk of malignant tumors and other diseases. Int. J. Mol. Sci. 21 (21), 7822. 10.3390/ijms21217822 33105665 PMC7659952

[B22] Rozmus D. Płomiński J. Augustyn K. Cieślińska A. (2022). rs7041 and rs4588 polymorphisms in vitamin D binding protein gene (VDBP) and the risk of diseases. Int. J. Mol. Sci. 23 (2), 933. 10.3390/ijms23020933 35055118 PMC8779119

[B23] Shang L. Li J. Zhou F. Zhang M. Wang S. Yang S. (2023). MiR-874-5p targets VDR/NLRP3 to reduce intestinal pyroptosis and improve intestinal barrier damage in sepsis. Int. Immunopharmacol. 121, 110424. 10.1016/j.intimp.2023.110424 37315369

[B24] Sheng M. Zhang Y. Hou T. (2021). Relationship of single nucleotide polymorphisms and genetic susceptibility to sepsis. Zhonghua Wei Zhong Bing Ji Jiu Yi Xue 33 (5), 630–632. 10.3760/cma.j.cn121430-20200922-00641 34112308

[B25] Singer M. Deutschman C. S. Seymour C. W. Shankar-Hari M. Annane D. Bauer M. (2016). The third international consensus definitions for sepsis and septic shock (Sepsis-3). JAMA 315 (8), 801–810. 10.1001/jama.2016.0287 26903338 PMC4968574

[B26] Song G. G. Bae S. C. Lee Y. H. (2016). Vitamin D receptor FokI, BsmI, and TaqI polymorphisms and susceptibility to rheumatoid arthritis: a meta-analysis. Z Rheumatol. 75 (3), 322–329. 10.1007/s00393-015-1581-6 26358095

[B27] Tayel S. I. Soliman S. E. Elsayed H. M. (2018). Vitamin D deficiency and vitamin D receptor variants in mothers and their neonates are risk factors for neonatal sepsis. Steroids 134, 37–42. 10.1016/j.steroids.2018.03.003 29530503

[B28] Wang Y. Zhu J. DeLuca H. F. (2012). Where is the vitamin D receptor? Arch. Biochem. Biophys. 523 (1), 123–133. 10.1016/j.abb.2012.04.001 22503810

[B29] Xiao K. Zhang D. C. Hu Y. Song L. C. Xu J. Q. He W. X. (2022b). Potential roles of vitamin D binding protein in attenuating liver injury in sepsis. Mil. Med. Res. 9 (1), 4. 10.1186/s40779-022-00365-4 35057868 PMC8772176

[B30] Xiao L. Que S. Mu L. Zheng R. (2022a). The relationship between vitamin D receptor gene and TREM-1 gene polymorphisms and the susceptibility and prognosis of neonatal sepsis. J. Clin. Lab. Anal. 36 (5), e24405. 10.1002/jcla.24405 35358332 PMC9102495

[B31] Yang X. Ru J. Li Z. Jiang X. Fan C. (2022). Lower vitamin D levels and VDR FokI variants are associated with susceptibility to sepsis: a hospital-based case-control study. Biomarkers 27 (2), 188–195. 10.1080/1354750X.2021.2024598 35001797

[B32] Yoo J. W. Jung Y. K. Ju S. Lee S. J. Cho Y. J. Jeong Y. Y. (2020). Serum vitamin D binding protein level, but not serum total, bioavailable, free vitamin D, is higher in 30-days survivors than in nonsurvivors with sepsis. Med. Baltim. 99 (25), e20756. 10.1097/MD.0000000000020756 PMC7310855 32569219

[B33] Zeljic K. Elkilany A. Supic G. Surbatovic M. Djordjevic D. Magic Z. (2017). Vitamin D receptor gene polymorphisms association with the risk of sepsis and mortality. Int. J. Immunogenet 44 (3), 129–134. 10.1111/iji.12318 28406554

